# RUNX1-mediated repression of miR-24 promotes hepatic stellate cell activation and liver fibrosis by targeting the ALK4/Smad3 signaling pathway

**DOI:** 10.3389/fgene.2026.1825784

**Published:** 2026-04-30

**Authors:** Zhijing Wang, Meiyi Song, Yingying Zhao, Jing Li, Lu Xia, Fei Wang, Changqing Yang

**Affiliations:** 1 Department of Gastroenterology and Hepatology, Tongji Hospital, School of Medicine, Tongji University, Shanghai, China; 2 Department of Gastroenterology, The First Affiliated Hospital of Shandong First Medical University and Shandong Provincial Qianfoshan Hospital, Jinan, China; 3 School of Biological Sciences, Jinan University, Guangzhou, China

**Keywords:** activin response-like kinase 4, hepatic stellate cell, liver fibrosis, miR-24, RUNX1

## Abstract

**Background:**

Epigenetic mechanisms, including microRNAs (miRNAs), are increasingly recognized as crucial regulators of organ fibrosis. In this study, we investigated the role of miR-24 in hepatic stellate cell (HSC) activation and liver fibrosis.

**Methods:**

miR-24 expression was analyzed in carbon tetrachloride (CCl_4_)-induced liver fibrosis and activated HSCs using quantitative real-time PCR (qRT-PCR). Gain- and loss-of-function experiments of miR-24 were performed *in vitro*. Western blotting, qRT-PCR, 5-ethynyl-2′-deoxyuridine (EdU) staining, flow cytometry, luciferase reporter assays, bioinformatics analysis, and chromatin immunoprecipitation PCR (ChIP-PCR) were performed to examine the molecular mechanisms of miR-24. Serum miR-24 levels were measured in patients with liver cirrhosis and further analyzed by subgroup.

**Results:**

We observed significant downregulation of miR-24 in CCl_4_-induced liver fibrosis and activated HSCs. Functional assays showed that miR-24 overexpression markedly inhibited HSC activation and migration, whereas miR-24 inhibition had the opposite effects. Mechanistically, ALK4 was identified as a direct target of miR-24: miR-24 bound the 3′UTR of ALK4 mRNA, thereby suppressing Smad3 phosphorylation and downstream fibrosis-associated signaling pathways. Furthermore, the transcription factor RUNX1 was induced during HSC activation, and it transcriptionally repressed miR-24 expression. Clinically, serum miR-24 levels were significantly lower in patients with liver cirrhosis than in healthy controls and were negatively correlated with Child–Pugh grade.

**Conclusion:**

Our findings suggest that the RUNX1/miR-24/ALK4 axis plays a crucial role in HSC activation and migration. miR-24 may serve as a biomarker for liver fibrosis screening, representing a potential therapeutic target for anti-fibrotic intervention.

## 1 Introduction

Liver fibrosis is a common pathological complication of various chronic liver diseases. Liver cirrhosis remains a major global health challenge and one of the leading causes of liver-related morbidity and mortality worldwide (Devarbhavi et al., 2023; Huang et al., 2023). Although age-standardized mortality has decreased in some settings, the absolute burden of cirrhosis and chronic liver disease continues to increase globally (Huang et al., 2023; Younossi et al., 2023). The occurrence of liver fibrosis is a long-term process. Chronic hepatitis virus infection, drug-induced liver injury, and hereditary metabolic diseases can converge on a similar core pathogenic pathway (Xu et al., 2017; Seitz et al., 2018; Asrani et al., 2019). In addition to liver biopsy, noninvasive tools such as serum-based indices and elastography are increasingly used for fibrosis staging diagnosis (Zhu et al., 2026). Meanwhile, emerging antifibrotic therapies, including resmetirom for MASH-related fibrosis, highlight the need for novel mechanistic and minimally invasive biomarkers (Le et al., 2025). Liver fibrosis is currently considered a reversible pathological stage. Regulation of hepatic stellate cells (HSCs), extracellular matrix (ECM), cytokines, and peptides is central to liver fibrosis and forms the basis for corresponding treatment options (Cordero-Espinoza and Huch, 2018; Molina et al., 2018). Excessive accumulation of ECM components in response to stimulation arises mainly from activated HSCs, predominantly collagens I and III (Pan et al., 2025). HSCs are also the main effector cells for cytokines such as transforming growth factor-β1 (TGF-β1) and platelet-derived growth factor (PDGF) that are induced during the initiation phase of fibrosis (Lee et al., 2015; Wilhelm et al., 2016; Wang et al., 2023). Subsequently, HSC signal transduction involves TGFβ1–Smad, cAMP–PKA–pCREB, and other intracellular pathways that contribute to the production of a cell-activated phenotype (Wang et al., 2015; Feng et al., 2025).

Epigenetic regulation enables rapid responses to the infectious stimuli within the complex intracellular network that drives HSC activation (Wilson et al., 2017). MicroRNAs (miRNAs, miRs) are endogenous small noncoding RNAs that function as essential epigenetic regulators. miRNAs implicated in liver fibrosis include miR-214, miR-499a-5p, miR-190b-5p, miR-296-3p, and miR-223 (Markovic et al., 2025; Wang et al., 2025; Zhao et al., 2025; Zhang et al., 2026). miR-24 is expressed across multiple species. In humans, miR-24-coding regions are located on chromosomes 9 and 19, forming a genetic cluster with miR-23 and miR-27b (Sharma et al., 2021). In mice, miR-24-1 and miR-24-2 are encoded on chromosomes 8 and 13, respectively. miR-24-1 and miR-24-2 are cleaved to generate a single mature miR-24-3p (Su et al., 2023). miR-24 plays a multifaceted role in modulating inflammation and cellular processes, including proliferation, development, and differentiation (Geng et al., 2016). However, the relationship between miR-24 and the biological behavior of HSC, as well as its regulatory mechanisms, remains unclear.

In the present study, we show that miR-24 is decreased in the fibrotic liver and activated HSCs. Overexpression of miR-24 in HSCs ameliorated the phenotypes of HSC activation during fibrosis, including proliferation, α-SMA- and ECM-related gene expression, and migration, by targeting activin receptor-like kinase 4 (ALK4), whereas miR-24 inhibition had the opposite effect. Additionally, we identified specific RUNX1 recognition sequences in the promoter of miR-24 and found that RUNX1-mediated repression of miR-24 acts as a critical molecular switch that promotes HSC activation and subsequent development of fibrotic characteristics, effectively bridging upstream transcriptional control with downstream effector signaling. Finally, miR-24 was decreased in the serum of patients with liver cirrhosis. Taken together, these findings indicate that miR-24 is a valuable anti-fibrotic miRNA in the liver and may represent a diagnostic marker and therapeutic target for liver fibrosis and cirrhosis.

## 2 Materials and methods

### 2.1 Establishment of the mouse liver fibrosis model

Eight-week-old male C57BL/6 mice were purchased from Cavens Laboratory (Changzhou). Carbon tetrachloride (CCl_4_) and olive oil were mixed at 1:5 (v:v). Mice were injected with CCl_4_ (2 mL/kg) twice a week for 6 weeks (12 times in total) and were sacrificed 2 days after the final CCl_4_ injection. All animals were fed a standard diet and kept under 12-h dark/light cycles at 21 °C–25 °C. Experimental protocols were approved by the Animal Ethics Committee of Shanghai Tongji Hospital (No. 20230101-DW-026).

### 2.2 Histological staining

Liver tissues were fixed in 4% paraformaldehyde (PFA), embedded in paraffin, and sectioned into 4 μm slices. Hematoxylin–eosin (H&E) and Sirius red staining were then performed according to the manufacturer’s protocol (Servicebio).

### 2.3 Cell culture and treatment

The rat HSC line HSC-T6 was provided by Jiangsu KeyGEN BioTECH Co., Ltd. HEK293T was purchased from the Chinese Academy of Sciences Cell Resource Center. BRL-3A was provided by Wuhan Pricella Biotechnology Co., Ltd. Cells were cultured in an incubator at 37 °C, 5% CO_2_, and saturated humidity. Cells were treated with a medium containing 10 ng/mL TGF-β1 (PeproTech) for 24 h.

### 2.4 Isolation of primary mouse hepatic stellate cell

High-purity primary HSCs were isolated from the livers of wild-type C57BL/6 mice using portal vein perfusion and digestion procedures (Mederacke et al., 2015). After anesthesia, the liver was removed and shredded after digestion with IV collagenase through portal vein perfusion. The tissue was further digested with DNase (0.07 g/L) and collagenase. The supernatant was collected by low-speed centrifugation at 40 g. Density gradient centrifugation using Nycodenz was conducted as the final step. Primary HSCs were counted, collected, and seeded in plastic plates at 2 × 10^5^/mL. Primary HSCs were cultured for up to 15 days to induce spontaneous activation.

### 2.5 miRNA mimic/inhibitor and siRNA transfection

Cells were seeded 1 day before transfection at 2 × 10^5^/mL. The final concentrations of miRNA mimic, miRNA inhibitor, and small interfering RNA (siRNA, Ribobio) were 50 nM, 100 nM, and 100 nM, respectively. For each well, 5 μL Lipofectamine 2000 (Invitrogen), siRNA/miRNA, and 200 μL Opti-MEM were mixed and incubated for 20 min. The mixture was added to cells with an additional 800 μL DMEM. Follow-up testing was performed 48 h after transfection.

### 2.6 Cell proliferation assay

Cell proliferation was detected using the 5-ethynyl-2′-deoxyuridine (EdU) cell proliferation detection kit (Ribobio). Cells were preincubated with EdU before the end of treatment (50 μM for 2 h in HSC-T6). Cells were fixed with 4% paraformaldehyde, permeabilized with 0.5% Triton X-100, and stained with Apollo solution according to the manufacturer’s instructions. Hoechst 33342 was used to stain cell nuclei. The percentage of EdU-positive cells among total nuclei was used to quantify cell proliferation.

### 2.7 Flow cytometric analysis of the cell cycle

Cells were digested with trypsin to prepare cell suspensions. We added −20 °C precooled absolute ethanol to the cell suspension and fixed it at −20 °C. The next day, cells were stained with propidium iodide (Sigma) for 15 min, and fluorescence intensity was measured using BD flow cytometry. Cell cycle distribution was analyzed using FlowJo 7.6.

### 2.8 Western blotting

Total protein from cells or tissues was extracted using cell lysis buffer. SDS–polyacrylamide gel electrophoresis was performed, and then, proteins were transferred to polyvinylidene fluoride (PVDF) membranes by wet transfer. Primary antibodies included anti-ALK4 (Abclonal), anti-Smad family member 3 (Smad3) (CST), anti-p-Smad3 (CST), anti-TGF-β1 (CST), anti-RUNX1 (Abclonal), anti-α-SMA (Abclonal), anti-Col1a1 (Bioworld), and anti-GAPDH (Bioworld). The next day, secondary antibodies were added and incubated at room temperature for 2 h. We developed the sample using the enhanced chemiluminescence (ECL) detection reagent.

### 2.9 Serum ALT and AST test

Before mice were sacrificed, whole blood was taken from the orbital sinus, and serum was separated. Serum alanine aminotransferase (ALT) and aspartate aminotransferase (AST) were measured using the Reitman–Frankel assay with an ALT/AST assay kit (Nanjing Jiancheng) according to the manufacturer’s instructions. ALT/AST concentrations for each sample were calculated from the standard quasi-curve.

### 2.10 Quantitative real-time PCR

Total RNA was isolated from cells or tissues using TRIzol. miRNA primers were purchased from Ribobio Biotechnology Company, and primers for other mRNAs are listed in Supplementary Table S1. cDNA was synthesized using a First Strand cDNA Synthesis Kit (Bio-Rad) according to the manufacturer’s instructions. qRT-PCR was performed using SYBR Green (TaKaRa). Relative expression was calculated using 2^−ΔΔCT^.

### 2.11 Transwell assay

Cells were collected and seeded into the upper chamber of polycarbonate membranes with 8-μm pores (Corning) at 100,000 cells/well. The lower chamber was filled with DMEM containing 10 ng/mL TGF-β1. After 24 h at 37 °C, membranes were stained with methylrosanilinium chloride solution according to the manufacturer’s recommendations (KeyGEN). Migrated HSCs that passed through the membrane into the lower chamber were counted using ImageJ from microscope images.

### 2.12 Luciferase reporter assay

To direct binding of miR-24 to ALK4, the 3′ untranslated region (UTR) of ALK4 was cloned into the luciferase reporter PGL3-basic vector (Promega). HEK293T cells were co-transfected with ALK4 3′UTR (or PGL3) and miR-24 mimic (or NC mimic). The activities of firefly and Renilla luciferase were assessed using a Dual-Luciferase Reporter Assay Kit (Promega) according to the manufacturer’s instructions.

### 2.13 ChIP-PCR

Chromatin immunoprecipitation was performed using the Pierce™ Magnetic ChIP Kit (Thermo Fisher Scientific). Cells were fixed in 1% formaldehyde, and the reaction was terminated by adding glycine. Chromatin was digested with micrococcal nuclease. Fragmented DNA was incubated with control IgG or anti-RUNX1 antibody (Proteintech) at 4 °C overnight. Protein G magnetic beads were added to capture immune complexes. RUNX1 binding to the miR-24 promoter was quantified using ChIP-PCR. Primer sequences targeting the miR-24 promoter are provided in Supplementary Table S1.

### 2.14 Liver cirrhosis patients

All human investigations conformed to the Declaration of Helsinki and were approved by the Institutional Review Committee of Shanghai Tongji Hospital (K-2023-024). A total of 27 patients with liver cirrhosis and 30 healthy controls were enrolled. Informed consent was obtained from each participant.

### 2.15 Statistical analysis

Data are expressed as the mean ± standard deviation (SD). Statistical analyses were performed using GraphPad Prism 8.0 and SPSS 23.0. Mann–Whitney U-tests or Student’s t-tests were used for comparisons between two groups. One-way or two-way analysis of variance (ANOVA) was used to compare multiple groups. Receiver operating characteristic (ROC) curves were generated to assess the ability of miR-24 to discriminate between diseases. Correlations between miRNA and serum biomarkers were determined using Spearman’s rank correlation. *p* < 0.05 was considered statistically significant.

## 3 Results

### 3.1 miR-24 is decreased in the fibrotic liver and activated HSCs

**FIGURE 1:**
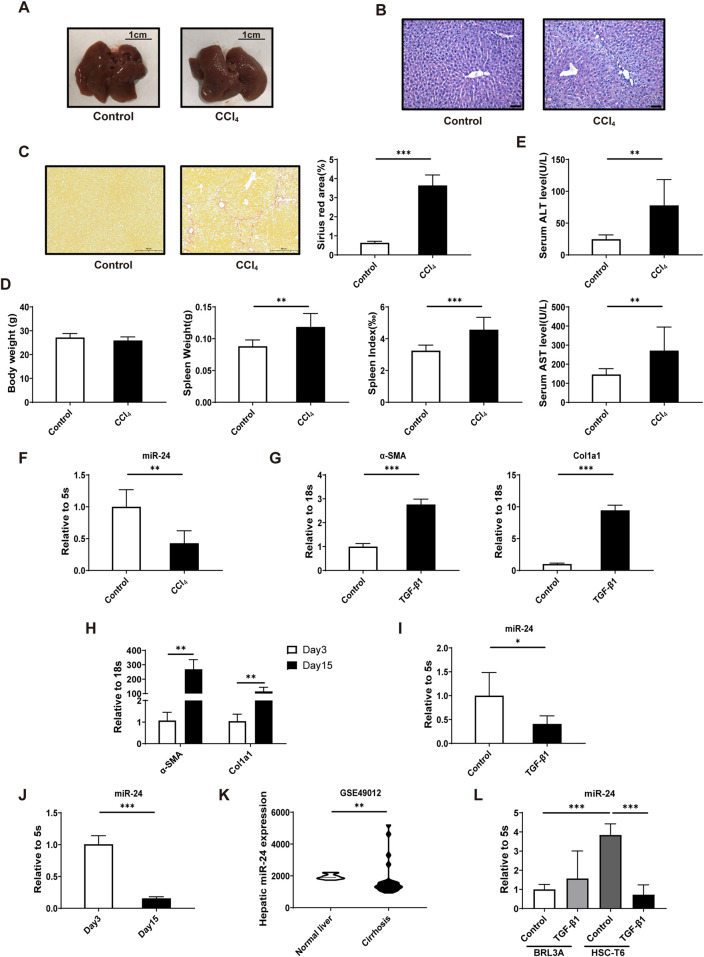
miR-24 is decreased in fibrotic livers and activated HSCs. **(A)** Representative images of mouse livers from control and CCl_4_-treated groups. Scale bar = 1 cm. **(B)** Representative H&E-stained images of mouse livers from control and CCl_4_-treated groups. Scale bar = 50 μm. **(C)** Representative Sirius red-stained images and quantification of mouse livers from control and CCl_4_-treated groups (n = 4:5). Scale bar = 200 μm. **(D)** Body weight, spleen weight, and spleen index of mice (n = 7:11). **(E)** Serum ALT and AST levels in mice (n = 7:11). **(F)** qRT-PCR analysis of miR-24 levels in liver samples from CCl_4_-induced mice (n = 3:8). **(G)** qRT-PCR analysis of α-SMA and Col1a1 expressions in HSC-T6s (n = 6 per group). **(H)** qRT-PCR analysis of α-SMA and Col1a1 expressions in activated primary mouse HSCs (n = 6 per group). **(I)** qRT-PCR analysis of miR-24 levels in HSC-T6 treated with TGF-β1 (n = 6 per group). **(J)** qRT-PCR analysis of miR-24 levels in activated primary mouse HSCs (n = 6 per group). **(K)** miR-24 expression in cirrhotic and healthy liver samples from published miRNA array data (GSE49012, n = 12:22). **(L)** qRT-PCR analysis of miR-24 levels in BRL-3A and HSC-T6 treated with TGF-β1 (n = 5:5:6:6). CCl_4_, carbon tetrachloride; HSC-T6, hepatic stellate cell-T6. Data are expressed as mean ± SD. *, *p* < 0.05; **, *p* < 0.01; ***, *p* < 0.001.

To clarify miR-24 expression in liver fibrosis, we built a murine liver fibrosis model via intraperitoneal injection of CCl_4_ 12 times. Mice were sacrificed 2 days after the final injection. Livers from CCl_4_-treated mice exhibited a granular surface, in contrast to the smooth morphology of the control group. H&E staining showed destruction of the hepatic lobule structure, with hepatocyte necrosis and mesenchymal hyperplasia, which are typical manifestations of liver fibrosis (Figures 1A,B). There was a marked increase in collagen fiber accumulation in the livers of CCl_4_-treated mice compared with the control group, as assessed using Sirius red staining (Figure 1C). There were no appreciable differences in body weight between control and CCl_4_-treated mice. The spleen weight and spleen index of the CCl_4_-treated group were higher than those of the control (Figure 1D). Serum ALT and AST were also significantly elevated in the CCl_4_-treated group (Figure 1E). We then used qRT-PCR to evaluate miR-24 expression in the liver and found that miR-24 was downregulated in the fibrotic liver (Figure 1F). HSCs are key contributors to liver fibrosis; therefore, we assessed changes in miR-24 expression during HSC activation. TGF-β1 treatment significantly promoted activation of HSC-T6 cells, as evidenced by elevated α-SMA and Col1a1 levels (Figure 1G). In addition, primary mouse HSCs were cultured for 15 days on plastic dishes to establish a culture activation model. Activation of primary HSCs was confirmed by increased α-SMA and Col1a1 (Figure 1H). Decreased miR-24 was also detected in these two activated HSC models (Figures 1I,J). We assessed hepatic miR-24 expression using published miRNA microarray data from healthy individuals and patients with liver cirrhosis (GEO: GSE49012) using GEO2R (Vuppalanchi et al., 2013). A decrease in miR-24 was detected in patients with liver cirrhosis (Figure 1K). In addition, miR-24 was relatively abundant in HSC-T6 cells. Upon TGF-β1 treatment, miR-24 levels decreased in HSCs but remained unchanged in BRL-3A hepatocytes (Figure 1L). These results indicate that miR-24 downregulation is a common hallmark of both the fibrotic liver and activated HSCs.

### 3.2 miR-24 regulates HSC activation

**FIGURE 2:**
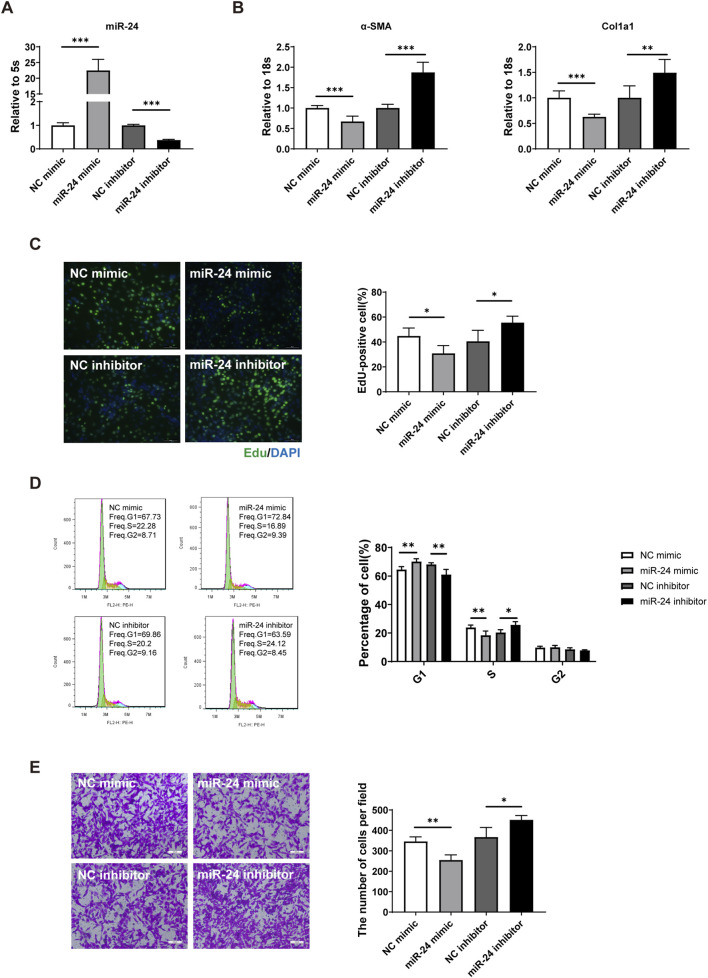
miR-24 influences HSC activation. **(A)** qRT-PCR analysis of miR-24 levels in HSC-T6 transfected with the miR-24 mimic or inhibitor (n = 6 per group). **(B)** qRT-PCR analysis of α-SMA and Col1a1 expressions in HSC-T6s (n = 6 per group). **(C)** EdU staining and quantification of EdU-positive HSC-T6s (n = 3 per group). Scale bar = 50 μm. **(D)** Flow cytometric analysis of the cell cycle distribution in HSC-T6s (n = 6 per group). **(E)** Transwell migration assay of HSC-T6s (n = 3 per group). Scale bar = 100 μm. HSC-T6, hepatic stellate cell-T6. Data are expressed as the mean ± SD. *, *p* < 0.05; **, *p* < 0.01; ***, *p* < 0.001.

Activated HSCs are a major source of ECM during liver fibrogenesis. Upon growth factor and cytokine stimulation, HSCs transdifferentiate from retinoid-storing cells into myofibroblasts and acquire features including cell growth, ECM turnover, contractility, and migration to sites of injury. To further investigate the effect of miR-24 on HSC activation, the rat HSC line HSC-T6 was transfected with the miR-24 mimic or inhibitor, and miR-24 expression was confirmed by qRT-PCR after 48 h of transfection (Figure 2A). We further investigated the function of miR-24 in HSC activation. Overexpression of miR-24 reduced α-SMA and Col1a1 expressions, while α-SMA and Col1a1 expressions were increased in the miR-24 inhibition group (Figure 2B). Primary HSCs isolated from the adult mouse liver were used to study the effects of miR-24 on the biological behavior of HSCs *in vitro*. Spontaneous activation of primary HSCs in culture is a model that mimics the transition from a quiescent to a myofibroblast phenotype observed *in vivo* during hepatic fibrogenesis. We found that miR-24 mimic reduced Col1a1 expression in primary HSCs, while miR-24 inhibition promoted Col1a1 expression (Supplementary Figure S1). The results demonstrated that miR-24 attenuates the increase in ECM synthesis typically observed during the spontaneous activation of primary HSCs, consistent with the findings in HSC-T6 cells. The proportion of Edu-positive cells declined after transfection with miR-24 mimic, whereas inhibition of miR-24 increased the proportion of Edu-positive cells (Figure 2C). Flow cytometric analysis showed that miR-24 mimic induced cell-cycle arrest at the G0/G1 phase in HSCs, whereas the miR-24 inhibitor had the opposite effect, promoting cell-cycle progression (Figure 2D). We further assessed whether miR-24 affects HSC migration. The transwell migration assay revealed that miR-24 overexpression significantly reduced the number of HSCs migrating through the polycarbonate membrane, whereas miR-24 inhibition increased migratory capacity (Figure 2E). Collectively, these findings suggest that miR-24 exerts an important role in HSC activation by regulating proliferation, ECM synthesis, and migration. Furthermore, restoration of miR-24 levels effectively attenuates the activation of HSCs.

### 3.3 ALK4 acts as a direct target gene of miR-24 in regulating HSC activation

**FIGURE 3:**
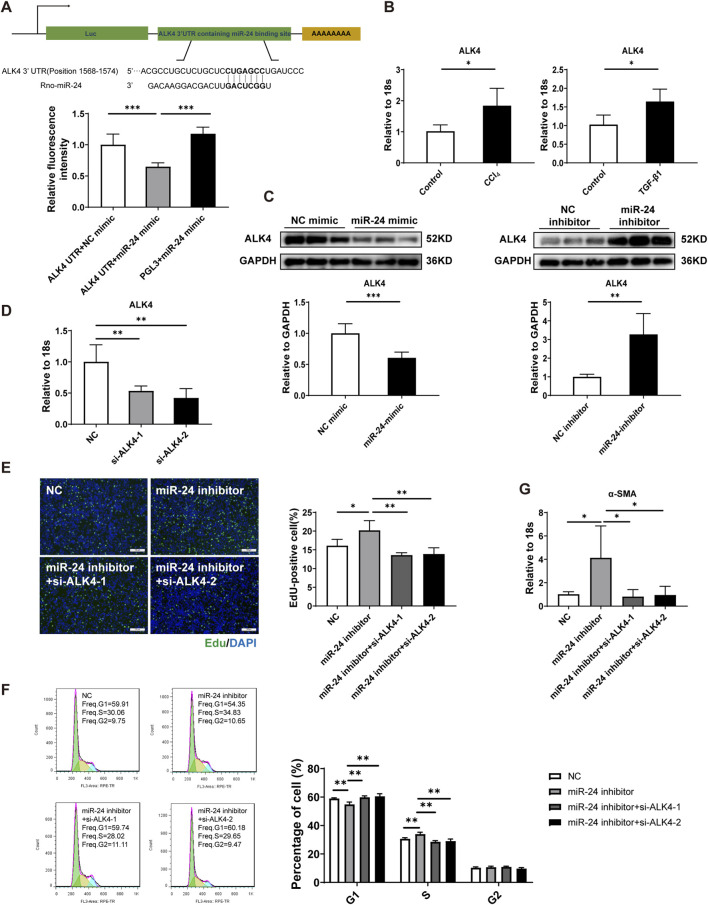
miR-24 regulates HSC activation by directly targeting ALK4. **(A)** Luciferase assay performed by transfection with the miR-24 mimic and interaction site on ALK4 3′UTR (n = 6 per group). **(B)** qRT-PCR analysis of ALK4 expression in CCl_4_-induced fibrotic livers and TGF-β1-induced HSC-T6s (n = 5 per group). **(C)** Western blot analysis of ALK4 expression in HSC-T6s (n = 6 per group). **(D)** qRT-PCR analysis of ALK4 expression in HSC-T6s (n = 6 per group). **(E)** EdU staining and quantification of EdU-positive HSC-T6s (n = 5 per group). Scale bar = 100 μm. **(F)** Flow cytometric analysis of the cell cycle distribution in HSC-T6s (n = 5 per group). **(G)** qRT-PCR analysis of α-SMA expression in HSC-T6s (n = 5:4:5:4). HSC-T6, hepatic stellate cell-T6. Data are expressed as the mean ± SD. *, *p* < 0.05; **, *p* < 0.01; ***, *p* < 0.001.

miRNAs bind seed sequences within the 3′UTR of target gene mRNAs to silence expression. Next, we aimed to identify direct target genes regulated by miR-24 in activated HSCs. ALK4 was reported to be a target gene regulated by miR-24 during erythroid differentiation (Wang et al., 2008). We first conducted a luciferase reporter gene assay, which showed that miR-24 bound to the 3′UTR of ALK4 at the GACUCGG site (Figure 3A). Moreover, ALK4 expression was increased in CCl_4_-induced liver fibrosis and activated HSCs, showing an opposing trend to miR-24 (Figure 3B). Western blotting showed that ALK4 was negatively regulated by miR-24 in HSCs (Figure 3C). We then performed a functional rescue experiment. siRNA-targeting ALK4 was used to knock down the expression of ALK4 in HSC-T6 (Figure 3D). We found that ALK4 inhibition eliminated the effects of miR-24 inhibition on the HSC activation phenotype. Importantly, the pro-proliferative effects induced by the miR-24 inhibitor were effectively abrogated by ALK4 siRNA transfection, as evidenced by a reduced proportion of EdU-positive cells and a decreased S-phase population (Figures 3E,F). ALK4 inhibition also reduced α-SMA mRNA expression induced by the miR-24 inhibitor (Figure 3G). Together, these results demonstrate that miR-24 suppresses HSC activation by directly targeting ALK4, identifying the miR-24/ALK4 axis as a critical regulator of hepatic fibrogenesis.

### 3.4 miR-24 inhibition promotes HSC activation via Smad3-dependent pathways

**FIGURE 4:**
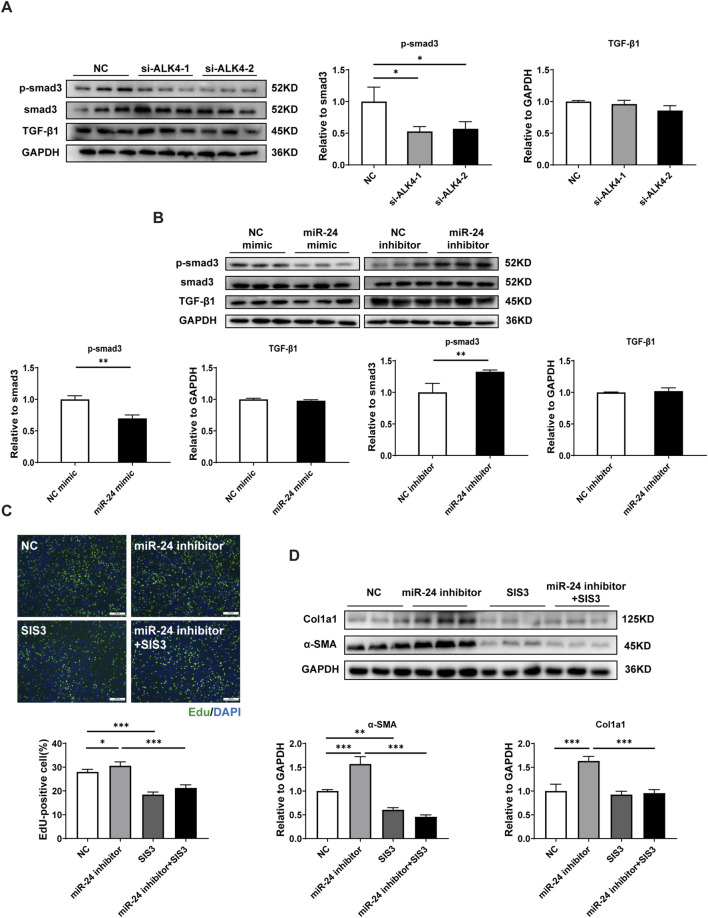
miR-24 suppression promotes HSC activation through Smad3-dependent mechanisms. **(A,B)** Western blot analysis of p-Smad3, Smad3, and TGF-β1 in HSC-T6s (n = 3 per group). **(C)** EdU staining and quantification of EdU-positive HSC-T6s (n = 6 per group). Scale bar = 100 μm. **(D)** Western blot analysis of α-SMA and Col1a1 in HSC-T6s (n = 3 per group). HSC-T6, hepatic stellate cell-T6. Data are expressed as the mean ± SD. *, *p* < 0.05; **, *p* < 0.01; ***, *p* < 0.001.

Smad3 is an important TGF-β1 signaling effector during liver fibrosis (Ding et al., 2013). Phosphorylated Smad3 forms a complex with Smad2 and Smad4 in the cytoplasm, then translocates to the nucleus, and activates effectors by recognizing Smad-binding elements (SBEs). ALK4, activated by activin or myostatin, can promote Smad3 phosphorylation (Mwaura et al., 2022). We found that Smad3 phosphorylation was significantly inhibited in ALK4-knockdown HSCs, whereas TGF-β1 expression was unaffected (Figure 4A). Similarly, miR-24 overexpression inhibited Smad3 phosphorylation in HSCs, whereas miR-24 inhibition enhanced Smad3 phosphorylation; TGF-β1 expression also remained unchanged following miR-24 modulation (Figure 4B). In addition, pharmacological inhibition of Smad3 phosphorylation by SIS3 significantly inhibited the activation phenotype triggered by the miR-24 inhibitor, suggesting that miR-24 regulates HSC activation in a Smad3-dependent manner (Figures 4C,D). Collectively, these findings indicate that miR-24 inhibition promotes HSC activation via Smad3-dependent pathways.

### 3.5 RUNX1-mediated transcriptional repression of miR-24 facilitates HSC activation

**FIGURE 5:**
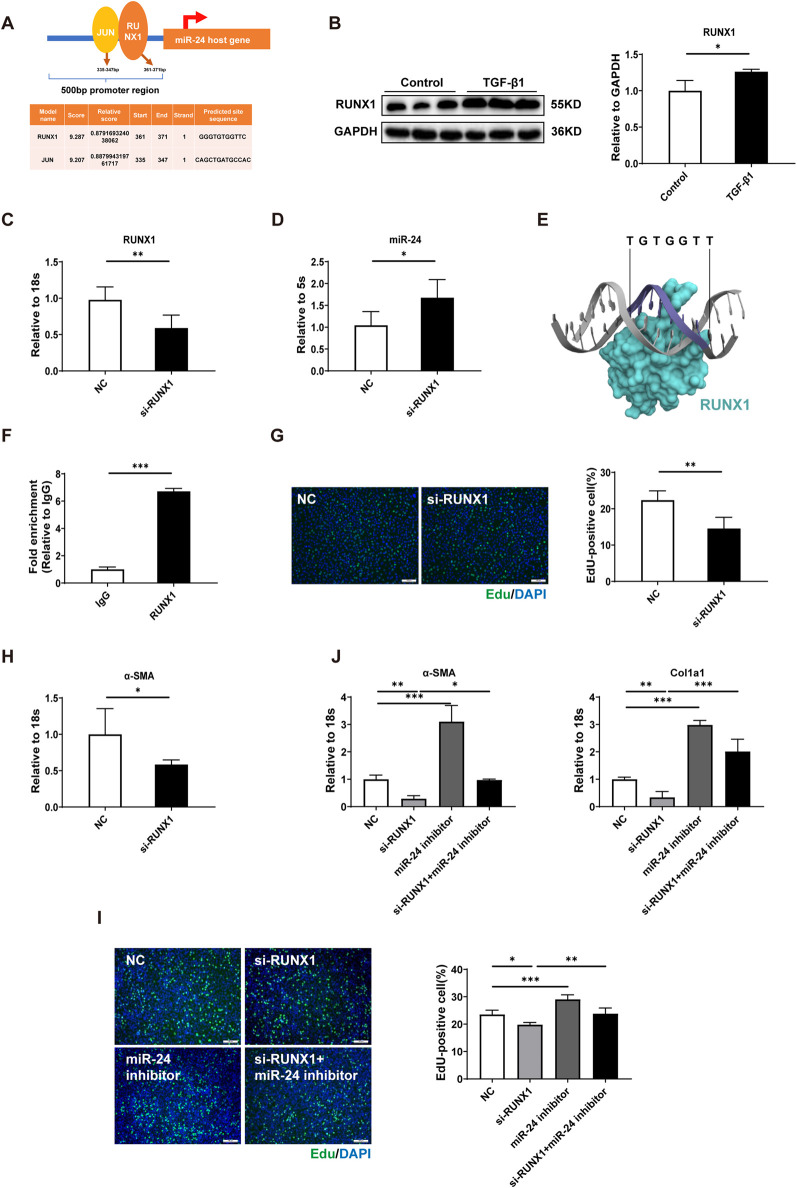
RUNX1 regulates miR-24 generation during HSC activation. **(A)** Promoter region analysis of the miR-24 host gene. **(B)** Western blot analysis of RUNX1 in HSC-T6s (n = 3 per group). **(C)** qRT-PCR analysis of RUNX1 expression in HSC-T6s (n = 6 per group). **(D)** qRT-PCR analysis of miR-24 expression in HSC-T6s (n = 6 per group). **(E)** Interaction between RUNX1 and the miR-24 promoter motif (PDB ID: 1HJC). **(F)** ChIP assay to determine RUNX1 enrichment at the miR-24 promoter in HSC-T6s (n = 3 per group). **(G)** EdU staining and quantification of EdU-positive HSC-T6s (n = 6 per group). Scale bar = 100 μm. **(H)** qRT-PCR analysis of α-SMA expression in HSC-T6s (n = 6 per group). **(I)** EdU staining and quantification of EdU-positive HSC-T6s (n = 5 per group). Scale bar = 100 μm. **(J)** qRT-PCR analysis of α-SMA and Col1a1 expressions in HSC-T6 cells (n = 5:5:3:3). HSC-T6, hepatic stellate cell-T6. Data are expressed as mean ± SD. *, *p* < 0.05; **, *p* < 0.01; ***, *p* < 0.001.

After characterizing the function and targets of miR-24, we aimed to identify factors responsible for the decrease in miR-24 during fibrogenesis, thereby uncovering upstream regulation of its expression. We queried the promoter sequence of miR-24 online using UCSC (http://genome-asia.ucsc.edu) and identified putative promoter motifs using Jasper (http://jaspar.genereg.net/). JUN and RUNX1 were selected based on the relative score (Figure 5A). C-JUN and RUNX1 were increased in activated HSCs induced by TGF-β1 (Figure 5B; Supplementary Figure S2A). By knocking down RUNX1 and JUN separately using siRNA, we observed that silencing RUNX1 significantly increased miR-24 expression, whereas JUN knockdown had no detectable effect, suggesting that RUNX1, but not JUN, acts as a negative regulator of miR-24 transcription (Figures 5C,D; Supplementary Figure S2B). Our molecular docking analysis based on the crystal structure of the RUNX1–DNA complex (PDB ID: 1HJC) indicated that RUNX1 recognizes the 5′-TGTGGT-3′ motif in the miR-24 promoter with high fidelity (Figure 5E). The ChIP assay confirmed direct binding of RUNX1 to the promoter region of miR-24 (Figure 5F). RUNX1 inhibition reduced proliferation and α-SMA mRNA expression (Figures 5G,H). To further establish the role of RUNX1 in regulating the miR-24-mediated HSC behavior, we conducted a functional rescue experiment. RUNX1 inhibition reduced proliferation and α-SMA and Col1a1 mRNA expression in HSCs, and this effect was reversed by miR-24 inhibition (Figures 5I,J). Collectively, these results suggest that the role of miR-24 in HSC activation is regulated by RUNX1.

### 3.6 Low-expression of serum miR-24 is detected in patients with liver cirrhosis

**FIGURE 6:**
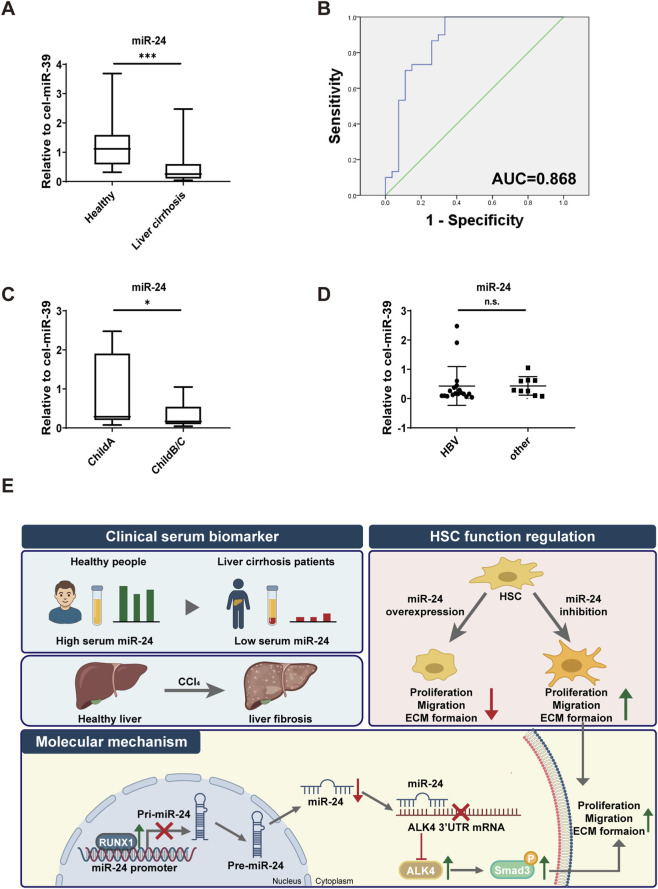
Downregulation of serum miR-24 in clinical liver cirrhosis. **(A)** qRT-PCR analysis of serum miR-24 in healthy controls and patients with liver cirrhosis (n = 30:27). **(B)** ROC curve showing the diagnostic performance of miR-24 for liver cirrhosis (n = 30:27). **(C)** qRT-PCR analysis of serum miR-24 in patients with liver cirrhosis stratified by the Child–Pugh grade (n = 7:20). **(D)** qRT-PCR analysis of serum miR-24 in patients with liver cirrhosis stratified by etiology (n = 18:9). **(E)** Working model of the RUNX1-miR-24-ALK4-Smad3 axis in liver fibrosis. HBV, hepatitis B virus. Data are expressed as the mean ± SD. n.s., not significant; *, *p* < 0.05; ***, *p* < 0.001.

Finally, we investigated whether miR-24 might serve as a serum marker for the diagnosis of liver cirrhosis. We enrolled 57 participants, including 30 healthy controls and 27 patients with liver cirrhosis. Baseline characteristics are listed in Table 1. Serum levels of miR-24 were measured in both groups by qRT-PCR. Serum miR-24 was significantly decreased in patients with liver cirrhosis compared with that in healthy controls (Figure 6A). The diagnostic potential of circulating miR-24 in liver cirrhosis was assessed using ROC analysis. An area under the curve value of 0.868 was achieved, indicating robust ability to identify cirrhotic individuals with high sensitivity and specificity (Figure 6B). Serum miR-24 was significantly lower in the Child–Pugh B or C group compared to Child–Pugh A patients, whereas no significant difference was observed among patients with cirrhosis caused by different etiologies (Figures 6C,D). No significant correlation was observed between serum miR-24 levels and biochemical markers, including hemoglobin (HGB), white blood cell (WBC), platelet (PLT), total bilirubin (TB), albumin (ALB), ALT, AST, and prothrombin time (PT), in patients with liver cirrhosis (Supplementary Table S2).

**TABLE 1:** Baseline characteristics of enrolled subjects.

Characteristic	Healthy (n = 30)	Liver cirrhosis (n = 27)
Age	42.57 ± 4.38	43.52 ± 7.66
ALT (U/L)	32.57 ± 20.14	68.48 ± 158.90
AST (U/L)	24.10 ± 8.09	73.63 ± 132.02***
ALB (g/L)	47.08 ± 3.08	30.80 ± 7.74***
TB (μmol/L)	15.74 ± 6.69	45.18 ± 49.31**
WBC (×10^9^/L)	6.77 ± 1.70	8.99 ± 13.58**
PLT (×10^9^/L)	240.77 ± 52.59	90.15 ± 73.27***

** *p* < 0.01

*** *p* < 0.001.

ALT, alanine aminotransferase; AST, aspartate aminotransferase; ALB, albumin; TB, total bilirubin; WBC, white blood cell; PLT, platelet.

## 4 Discussion

Liver fibrosis is a reversible pathological stage that precedes liver cirrhosis. In the current study, we provide evidence that miR-24 functions as a suppressor of liver fibrosis. We demonstrate that RUNX1–miR-24–ALK4 signaling regulates HSC activation via Smad3 signaling. In addition, miR-24 may serve as a biomarker in patients with liver cirrhosis (Figure 6E).

The pathogenesis of hepatic fibrosis involves diverse profibrogenic factors, cells, and immunomodulators. miRNAs are essential components of epigenetic regulation in fibrogenesis; for example, miR-122 suppresses the proliferation and activation of HSCs by targeting EphB2 (Ma et al., 2022). miR-223 suppresses neutrophil elastase (NE) and proteinase 3 (PR3) by targeting the signal transducer and activator of transcription 3 (STAT3), thereby alleviating metabolic dysfunction-associated steatohepatitis (MASH) and liver fibrosis in mice (Zhang et al., 2026). Sodium butyrate intervention inhibited HSC activation via the miR-155-5p/suppressor of cytokine signaling 1/PDGF signaling pathway, thereby alleviating hepatic fibrosis of MASH (Huang et al., 2025). In the multidrug resistance gene-2 knockout mouse, miR-24 inhibition aggravates manifestations of primary sclerosing cholangitis, including hepatic fibrosis (Hall et al., 2017). LncRNA Gm5091 reduces miR-27b/23b/24 levels and alleviates the development of alcoholic hepatic fibrosis (Feng et al., 2017; Zhou et al., 2018). Human umbilical cord mesenchymal stem cell-derived exosomes alleviate MASH by regulating macrophage polarization via the miR-24-3p targeting stimulator of interferon genes (STING) (Jiang et al., 2025). Downregulation of miR-24 and miR-27a has been reported in hepatocellular carcinomas from cirrhotic liver tissues compared with that in non-cirrhotic liver tissues (Salvi et al., 2013). Similarly, miR-24 regulates cardiac fibrosis and muscle fibrosis as an anti-fibrotic miRNA (Sun et al., 2018; Zhang et al., 2022; Senesi et al., 2025). We found a significant reduction in miR-24 in CCl_4_-induced liver fibrosis and HSC activation. HSCs contribute 82%–96% of myofibroblasts during different types of chronic liver diseases (Mederacke et al., 2013) and are the principal source of collagen-rich ECM production (Hui et al., 2018; Inzaugarat et al., 2018). In our study, we used two widely accepted approaches to activate HSCs: TGF-β1-induced activation and culture-induced activation of primary HSCs (Tsuchida and Friedman, 2017; Du et al., 2018). We found that miR-24 was decreased in both activation models. To elucidate the role of miR-24 in liver fibrosis, we investigated how its dysregulation alters HSC activation. Our findings suggest that reduced miR-24 promotes myofibroblast transformation and excessive ECM production in HSCs. In parallel, miR-24 exerted a negative regulatory effect on HSC migration *in vitro*, providing further evidence for its involvement in liver fibrosis. Chemotaxis-induced migration is a hallmark of activated HSCs, whereas quiescent HSCs are typically sequestered within the Disse Space; upon activation, cells migrate and accumulate at the sites of injury (Yang et al., 2003; Ceni et al., 2017). Thus, miR-24 deficiency may act as a trigger for HSCs to adopt an activated, profibrotic state, characterized by increased matrix synthesis and cellular motility, which are essential for fibrogenesis. Depletion of circulating miR-24 was observed in cirrhotic patients, regardless of the underlying etiology. We suggest that miR-24 could serve as a promising, non-invasive biomarker for the diagnosis of liver cirrhosis.

ALK4, as a type I receptor for activin A, contains a ligand-binding domain that interacts with activin A. Activin A signal transduction occurs in two steps: activin type II receptors, which have high affinity, bind to activin A, and then ALK4 is recruited to the receptor complex (Zhu et al., 2012; Klauzinska et al., 2014). Smad proteins are shared downstream effectors of activin/ALK4 pathways and TGF-β1 in regulating liver fibrosis. Intracellular receptor-activated Smad2 and Smad3 form a complex with Smad4, and this complex translocates to the nucleus, recognizes SBE in the genome, and directly regulates target genes (Akhurst, 2012). Meanwhile, a reduction in ALK4 has been reported to alleviate myocardial infarction-induced cardiac fibrosis through Smad3/4 pathways (Chen et al., 2022). In the present study, we show that miR-24 directly targets the 3′UTR of ALK4, leading to post-transcriptional silencing. Consequently, miR-24 suppresses Smad3 phosphorylation independently of TGF-β1 levels. This mechanism confirms that miR-24 acts as a molecular brake on the ALK4/Smad3 axis, thereby inhibiting profibrogenic signaling. In addition, either blocking Smad3 phosphorylation or inhibiting ALK4 eliminated the promotion of HSC activation induced by miR-24 knockdown.

The primary driving forces underlying differential miRNA expression during fibrogenesis remain to be elucidated. RUNX1 is a hematopoietic transcription factor that contributes to angiogenic and vasculogenic phenotypes through interactions with other transcriptional regulators, including hypoxia-inducible factor-1α and insulin-like growth factor binding protein-3 (Harris et al., 2013). RUNX1 is a prototypical context-dependent transcription factor whose activity is dictated by the composition of transcriptional complexes recruited to specific genomic regions. Mechanistically, RUNX1-mediated recruitment of histone deacetylase 1 and TLE family corepressors contributes to enhancer repression and transcriptional silencing of target genes (Guo et al., 2020; Simeoni et al., 2021). RUNX1 is also identified as a UTE-1-binding protein and is induced at the post-transcriptional level during HSC activation in the liver (Bertrand-Philippe et al., 2004). Consistent with previous studies, we suggest that RUNX1 promotes proliferation and collagen synthesis in HSCs. We identified a RUNX1 binding site in the promoter region of the miR-24 host gene and observed upregulation of miR-24 upon RUNX1 inhibition in HSCs. We demonstrate that, under fibrotic conditions, RUNX1 favors the recruitment of repressive machinery, leading to transcriptional silencing of miR-24 through the modulation of promoter-proximal elements. This sequence of events—from epigenetic silencing to receptor-mediated signal amplification—highlights a regulatory network in which RUNX1 acts as a master switch in the transition of HSCs to an activated phenotype.

In conclusion, the present study highlights the potential therapeutic relevance of miR-24 in murine and human liver fibrosis. miR-24 functions as an anti-fibrotic regulator by post-transcriptionally targeting ALK4, thereby suppressing Smad3-mediated HSC activation and ECM deposition. The function of miR-24 in HSC activation is regulated by RUNX1. In addition, decreased serum miR-24 in patients with liver cirrhosis suggests a novel biomarker for liver cirrhosis.

## Data Availability

The original contributions presented in the study are included in the article/Supplementary Material; further inquiries can be directed to the corresponding authors.
