# Supplementary material for: RUNX1-mediated repression of miR-24 promotes hepatic stellate cell activation and liver fibrosis by targeting the ALK4/Smad3 signaling pathway
Source: Front Genet. 2026 Apr 30;17:1825784. doi: 10.3389/fgene.2026.1825784 (PMC13171067; doi:10.3389/fgene.2026.1825784)
Supplement: Supplementary file 1 [file DataSheet1.docx]

Supplementary Material

# Supplementary Figures and Tables

## Supplementary Figures


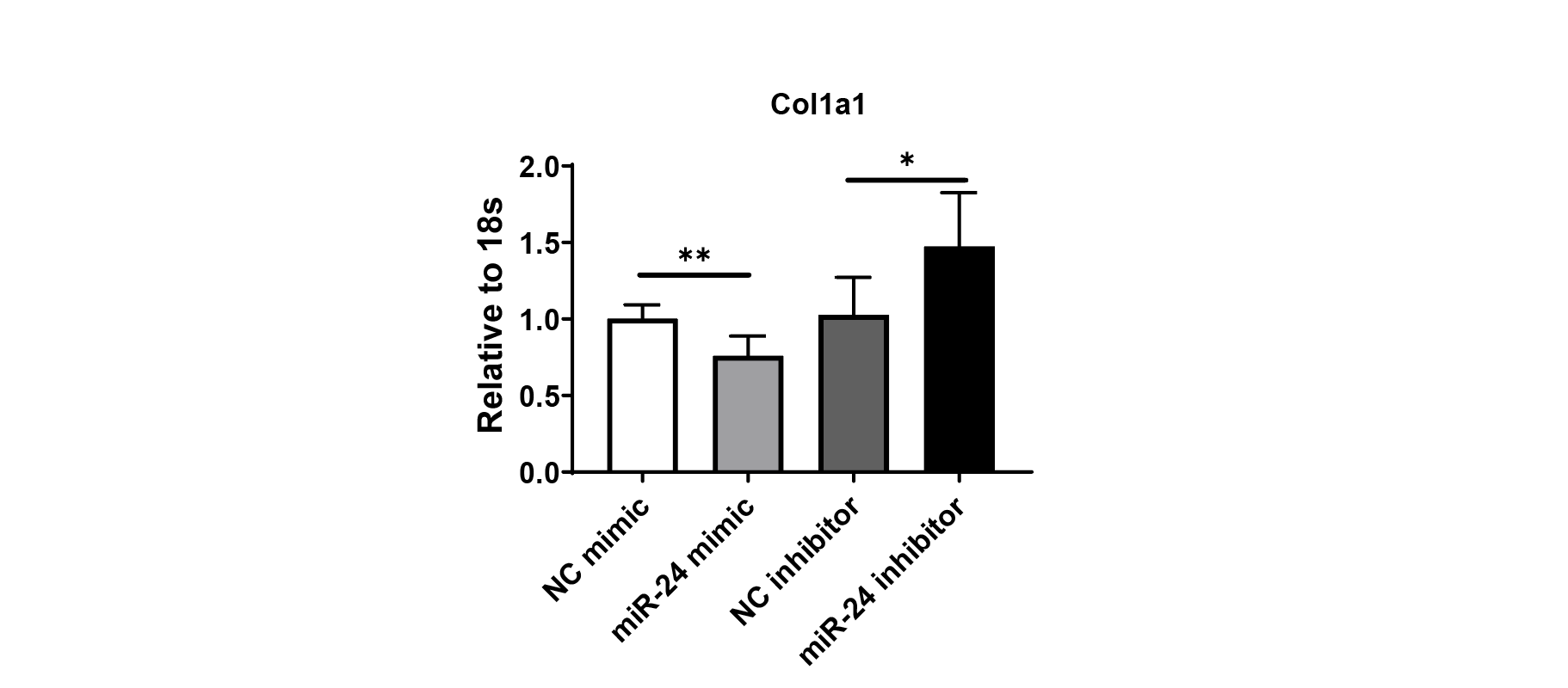


**Supplementary Figure 1.** miR-24 regulates the expression of Col1a1 in primary mouse HSCs. qRT-PCR analysis of Col1a1 expression in primary mouse HSCs (n=6 per group). Data are expressed as mean ± SD. *, P<0.05 and **, P<0.01.


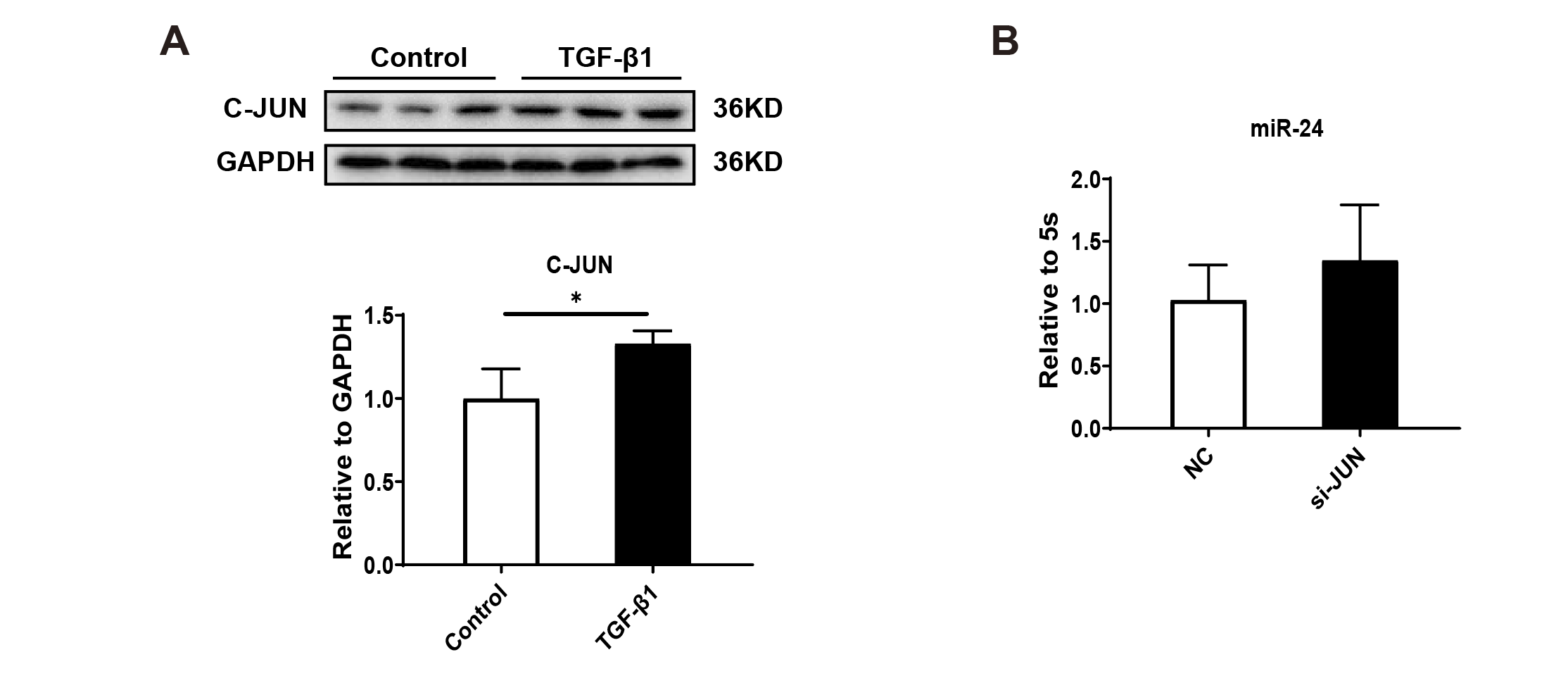


**Supplementary Figure 2.** miR-24 is not controlled by C-JUN in HSC activation. A.Western blot analysis of C-JUN in HSC-T6s (n=3 per group). B. qRT-PCR analysis of miR-24 expression in HSC-T6s (n=5 per group). HSC-T6, hepatic stellate cell-T6. Data are expressed as mean ± SD. *, P<0.05.

## Supplementary Tables

**Supplemental Table 1.** Oligonucleotide sequences used in this study

| **Gene** | **Primer sequence (5’-3’)** |
| --- | --- |
| rno-*18S*-Forward | TCAAGAACGAAAGTCGGAGG |
| rno-*18S* -Reverse | GGACATCTAAGGGCATCAC |
| rno-α-SMA-Forward | GTGATCACCATCGGGAATGA |
| rno-α-SMA -Reverse | CAGCAATGCCTGGGTACATG |
| rno-*COL1A1*-Forward | AACCCCAAGGAGAAGAAGCA |
| rno-*COL1A1*-Reverse | AGCGTGCTGTAGGTGAATCG |
| rno-Alk4-Forward | GGGATCCAGGCTCTGCTG |
| rno-Alk4-Reverse | ACTCAGGCAGTAGAAGGGCT |
| rno-Runx1-Forward | AAACTACTCGGCGGAGCTGAGAA |
| rno-Runx1-Reverse | TTGATGGCTCTATGGTAGGTGGCA |
| mmu-*18S*-Forward | ACCCGTTGAACCCCATTCGTGA |
| mmu-*18S* -Reverse | GCCTCACTAAACCATCCAATCGG |
| mmu-α-SMA-Forward | CATGTCGTCCCA GTTGGTGAT |
| mmu-α-SMA -Reverse | GTTTTGTGGATCAGCGCCTC |
| mmu-*COL1A1*-Forward | GAGCGGAGAGTACTGGATCG |
| mmu-*COL1A1*-Reverse | GCTTCTTTTCCTTGGGGTTC |
| mmu-Alk4-Forward | GGTCTTGGTTCCGTGAAGCAGA |
| mmu-Alk4-Reverse | GCTCGTGATAGTCAGAGACAAGC |
| ChIP-miR-24-Forward | CATCAAGGAAACTGAGCCAACCTT |
| ChIP-miR-24-Reverse | TACAAGAAAGAGTTCACCACGC |

**Supplemental Table 2.** Correlation between serum miR-24 levels and clinical biochemical parameters in patients with liver cirrhosis.

| **Parameters** | **P value** |
| --- | --- |
| HGB | 0.093 |
| WBC | 0372 |
| PLT | 0.780 |
| TB | 0.684 |
| ALB | 0.359 |
| ALT | 0.419 |
| AST | 0.255 |
| PT | 0.467 |

HGB, Hemoglobin; WBC, White Blood Cell; PLT, Platelet; TB, Total Bilirubin; ALB, Albumin; ALT, Alanine Aminotransferase; AST, Aspartate Aminotransferase; PT, Prothrombin Time.
